# Trends in antidiabetic drug use and expenditure in public hospitals in Northwest China, 2012-21: a case study of Gansu Province

**DOI:** 10.1186/s12913-024-10917-0

**Published:** 2024-04-03

**Authors:** Wenxuan Cao, Hu Feng, Yaya Yang, Lei Wang, Xuemei Wang, Yongheng Ma, Defang Zhao, Xiaobin Hu

**Affiliations:** 1 School of Public Health, Lanzhou University, 222# Tianshui South Road, Lanzhou, 730000 China; 2 Division of Pharmaceutical Procurement, Gansu Public Resources Trading Center, 68# Yanxing Road, Lanzhou, 730000 China

**Keywords:** Antidiabetic drug, Public hospitals, Use pattern, Pharmaceutical policy, China

## Abstract

**Background:**

Since the twenty-first century, the prevalence of diabetes has risen globally year by year. In Gansu Province, an economically underdeveloped province in northwest China, the cost of drugs for diabetes patients accounted for one-third of their total drug costs. To fundamentally reduce national drug expenditures and the burden of medication on the population, the relevant departments of government have continued to reform and improve drug policies. This study aimed to analyse long-term trends in antidiabetic drug use and expenditure in Gansu Province from 2012 to 2021 and to explore the role of pharmaceutical policy.

**Methods:**

Data were obtained from the provincial centralised bidding and purchasing (CBP) platform. Drug use was quantified using the anatomical therapeutic chemistry/defined daily dose (ATC/DDD) method and standardised by DDD per 1000 inhabitants per day (DID), and drug expenditure was expressed in terms of the total amount and defined daily cost (DDC). Linear regression was used to analyse the trends and magnitude of drug use and expenditure.

**Results:**

The overall trend in the use and expenditure of antidiabetic drugs was on the rise, with the use increasing from 1.04 in 2012 to 16.02 DID in 2021 and the expenditure increasing from 48.36 in 2012 to 496.42 million yuan in 2021 (from 7.66 to 76.95 million USD). Some new and expensive drugs changed in the use pattern, and their use and expenditure shares (as the percentage of all antidiabetic drugs) increased from 0 to 11.17% and 11.37%, but insulins and analogues and biguanides remained the most used drug class. The DDC of oral drugs all showed a decreasing trend, but essential medicines (EMs) and medical insurance drugs DDC gradually decreased with increasing use. The price reduction of the bid-winning drugs was over 40%, and the top three drugs were glimepiride 2mg/30, acarbose 50mg/30 and acarbose 100mg/30.

**Conclusions:**

The implementation of pharmaceutical policies has significantly increased drug use and expenditure while reducing drug prices, and the introduction of novel drugs and updated treatment guidelines has led to changes in use patterns.

## Background

About one in ten of the world's population is diabetes patient, and what's worse is that this percentage is increasing every year. According to the International Diabetes Federation, in 2021, 537 million adults (aged 20–79) worldwide had diabetes, resulting in an estimated total global healthcare expenditure of 966 billion USD [1]. China had the largest number of diabetes patients in the world. According to statistics, approximately 11.2% of adults aged 18 years and older had diabetes in 2021, with the total number of patients reaching 141 million, and it was expected to reach 174 million by 2045 [2].

Gansu Province is an economically underdeveloped province in northwestern China, and a study showed that the local diabetes prevalence rate was 7.33% in 2018 [3]. In the same year, the total healthcare cost of diabetes patients was 1.348 billion yuan (20.37 million USD) [4], accounting for 2.56% of the total treatment cost in the province and 0.16% of the regional gross domestic product (GDP). Although this percentage was not high, the cost of drugs accounted for about one-third, which was much higher than the average level of 19.7% among the member countries of the Organisation for Economic Co-operation and Development in 2019 [5].

Appropriate use of antidiabetic drugs is key in the management of diabetes (especially type 2 diabetes), which is important for delaying disease progression, reducing the risk of complications and reducing the disease burden. To fundamentally reduce national drug expenditure and the burden of drug use on the population, relevant Chinese government departments have been reforming and improving their drug policies. The National Essential Medicine System (NEMS) was first established in 2009, provinces and cities across the country have started to implement the "three unified" (policies of unified bidding, distribution, and pricing) and zero-price markup policy to expand the coverage of essential medicines (EMs) [6]. And all EMs were included in the National Basic Medical Insurance Drug Catalogue (NBMIDC), so their reimbursement rate was higher than that of non-EMs. As Chinese residents' basic medical insurance coverage was upwards of 95% [7], this policy significantly reduced out-of-pocket payment for residents.

Since the beginning of the three-year new medical reform (2009–11), Gansu has involved government-run medical and health institutions in the scope of the national EMs management and has achieved zero mark-up drugs——No mark-ups or other surcharges are allowed in the price of medicines, the actual selling price is close to the cost price. By the end of 2012, the coverage rate of the EMs system reached 100%. After the policy came to maturity, it successfully reduced the drug cost share from 34.98% in 2014 to 26.91% in 2018 within four years [8]. At the same time actively implementing the national centralised drug procurement policy, until 2021, Gansu Province has carried out the national organization of five batches of 218 drugs centralized procurement work, which contained a total of 14 varieties (based on Anatomical Therapeutic Chemical (ATC) -5), 23 strains (based on formulations, dosage specifications and manufacturers) of antidiabetic drugs [9–13]. According to incomplete statistics from relevant government departments, the policy has led to an average price reduction of 54.6% for the drugs included and relative cost savings of more than 1.3 billion yuan (20.15 million USD) [14]. To date, several studies have reported on temporal trends in the use of antidiabetic drugs in other foreign countries, but few studies have focused on changes in the use and expenditure of this class of drugs in less economically developed areas of China and the role of pharmaceutical policies behind the changes [15, 16].

This study collected data on antidiabetic drugs in Gansu Province from 2012 to 2021, aiming (1) to explore the long-term trends and use patterns of antidiabetic drug use and expenditure in Northwest China, (2) to reveal the impact of the implementation of pharmaceutical policy on drug use and DDC.

## Methods

### Study setting

Gansu Province is in northwest China, with a narrow and curving topography, and is an economically underdeveloped province. The province's GDP was 102.43 billion yuan (15.88 billion USD) in 2021——the fifth lowest in the country [17]. With over 70% of the population living in rural, the dispersed nature of the population due to geography and economic level, limited medical resources and inadequate public health education were the main reasons that prevented diabetes patients in the region from effectively managing their disease [18]. The residents' health insurance coverage was over 97%, and the patient's medical expenses were shared between the national health insurance (coordinated payment) and the patient (individual out-of-pocket payment) within reasonable reimbursement limits [19].

### Data source

The study used data from the centralised bidding and purchasing (CBP) platform managed by the Drug Procurement Division of the Gansu Provincial Public Resources Transaction Center. Its main record information included the drug’s generic name, dosage form, specification, conversion factor, approval number, manufacturer, purchasing unit, purchasing time, purchasing quantity and amount. By 2020, the number of hospitals covered by this database accounted for 93.39% of the total number of public hospitals. This study adopted a retrospective research method, based on the daily drug procurement data of public hospitals in the province from 2012 to 2021, to sort out a total of 2 major categories (based on ATC-3), 10 subcategories (based on ATC-4), 40 varieties (based on ATC-5) and 338 strains (based on formulations, dosage specifications and manufacturers) of antidiabetic drugs, basically covering the main types of diabetes therapeutic drugs. In China, all antidiabetic drugs approved by the healthcare authorities are prescription drugs, and diabetes patients must be prescribed them by clinicians and obtained from hospital pharmacies.

### Data management

The ATC system classified drugs into different groups according to the organ or system on which they act and chemical, pharmacological and therapeutic properties. Drugs were classified into ATC groups by its international non-proprietary name. In this paper, all "A10" (drugs used in diabetes) in "A" (alimentary tract and metabolism) were included in the study. Among them, we emphasized the use of a novel antidiabetic drug group, with the following ATC codes: A10BH and A10BD07-13 for dipeptidyl peptidase 4 inhibitors (DPP-4i); A10BJ, A10AE54 and A10AE56 for glucagon-like peptide-1 receptor analogue (GLP-1RAs); A10BK, A10BD15 and A10BD20 for sodium-glucose co-transporter 2 inhibitors (SGLT2i) [20].The data was analysed using the ATC/defined daily dose (DDD) system developed by the WHO [21], which calculated the frequency of use of defined daily doses (DDDs) and the defined daily cost (DDC) of each drug based on the DDD, and expressed the standardised dosing intensity in terms of doses per 1000 inhabitants per day (DID). The DDD is the average daily dose used in adults for the primary therapeutic purpose, based mainly on the DDD values of drugs published on the official website; DDDs and DDC were calculated using the following formula, the higher the DDDs, the more frequently such drugs were used and the greater the clinical tendency to choose the drug; the higher the DDC, the greater the financial burden on the patient.

$$\begin{array}{c}DDDs={\sum }_{i=1}^{n}(Unit\ strength\times Packing\ specification)/DDD\times {N}_{i}\\ DDC=\frac{Total\ drug\ expenditure}{DDDs}\end{array}$$

where *N*_*i*_ represents the number of packages of drug (*i*).

### Data analysis

The number of residents per year was expressed as the average of the population at the end of that year and the previous year in the Gansu Statistical Yearbook (mid-year population) [22], on the basis of which the number of permanent residents was adjusted using data on the mobile population in Gansu Province [23] in order to reduce any possible errors in the results that may result from this. Expenditure was adjusted for price inflation at an annual rate of 1.42% (the average annual inflation rate of the Gansu provincial consumer price index from 2012 to 2021) [24]. Descriptive statistics were used to describe the annual use and expenditure of antidiabetic drugs in Gansu Province, and to calculate the use shares (as the proportion of the total use) and DDC values of each drug type. Linear regression was used to analyse the changes in the use and expenditure of each type of drug during the study period (at least five consecutive years of purchase records), and the regression coefficients (*B* value) and significance (*P* value) were used to indicate the direction of the trend. Whether the *B* value is greater than 0 indicates the direction of change (> 0—upward, < 0—downward), and *P* values < 0.05 were taken to indicate statistical significance. Microsoft Office Excel 2016 and Stata 16.0 were used for data management and analysis, and GraphPad Prism 9 was used for graphing.

## Results

### Overview of the use of antidiabetic drugs

During the ten-year study period, the overall trend of antidiabetic drug use and expenditure in Gansu Province was increasing (*B* = 1.373, 44.229, *P* < 0.001), use increased from 1.04 in 2012 to 16.02 DID in 2021, and expenditure increased from 48.36 in 2012 to 496.42 million yuan in 2021 (from 7.66 to 76.95 million USD), with the largest increase in 2013 and the largest decrease in 2018.

**Fig. 1:**
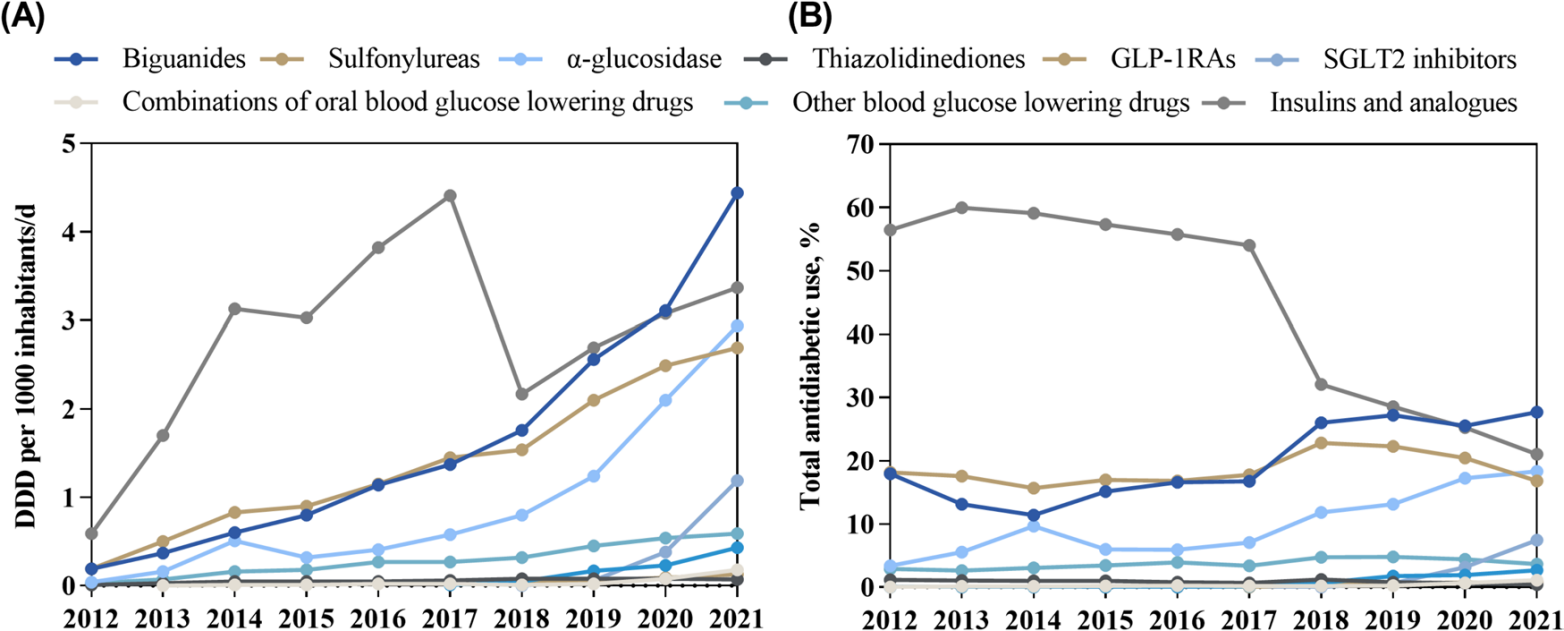
Trends in the use and proportion of antidiabetic drugs. **A** trends in the use of antidiabetic drugs; **B** trends in the proportion of antidiabetic drugs. GLP-1RAs, glucagon-like peptide-1 receptor analogues; SGLT2, sodium-glucose co-transporter 2; DDD, defined daily dose

There were large differences in the composition of use by drug class. Insulins and analogues were the most used, but their proportion of use decreased by almost 20% over the decade (*B* = -0.048, *P* < 0.001). In contrast, the proportion of use of oral antidiabetic drugs remained mostly stable or increased. For example, the proportion of use of biguanides increased by about 10% (*B* = 0.017, *P* = 0.002), the proportion of use of sulfonylureas remained stable at about 20%, followed by α-glucosidase inhibitors, its proportion of use increased rapidly to one-fifth of the total (*B* = 0.272, *P* = 0.001) (Fig. 1).

**Fig. 2:**
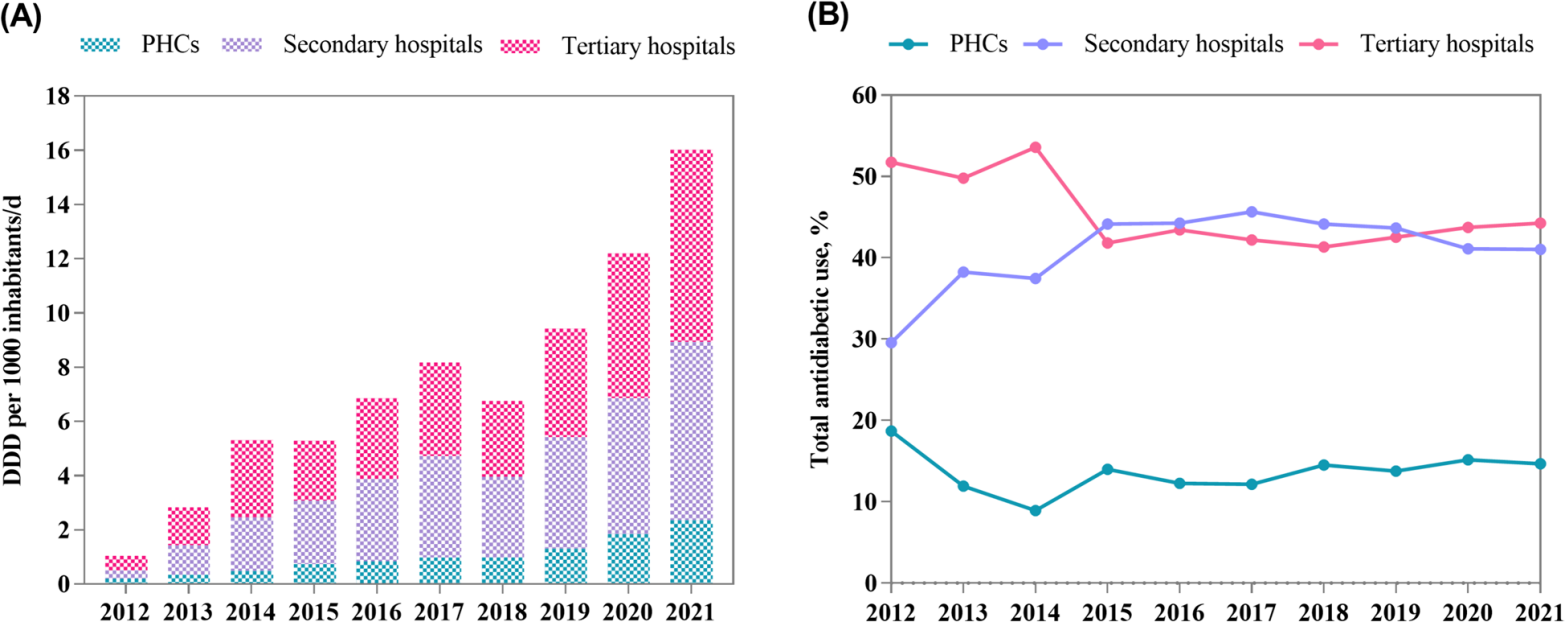
Trends in the use and proportion of antidiabetic drugs in different hospital levels. **A** trends in the use of antidiabetic drugs in different hospital levels; **B** trends in the proportion of antidiabetic drugs in different hospital levels. PHCs, primary health care centres; DDD, defined daily dose

Among the different hospital levels, the use was comparable between tertiary and secondary hospitals (44.19% vs 42.18%) and lowest in primary hospitals (13.63%). Drug use increased in all hospital levels during the study period but with a gradual decrease in the proportion of drugs used in tertiary hospitals (*B* = -0.010, *P* = 0.029) and an increasing trend in the proportion of drugs used in secondary (*B* = 0.009, *P* = 0.042) (Fig. 2).

### Trends in the use of novel antidiabetic drugs

The total use (*B* = 0.420, *P* = 0.034) and expenditure (*B* = 13.529, *P* = 0.008) of novel antidiabetic drugs have continued to increase since 2017, and their shares reached 11.17% and 11.37% of the total by 2021 respectively. The most widely used class of novel antidiabetic drugs was SGLT2i, accounting for 56.67% of the total use in this group, followed by DPP-4i and GLP-1RAs with 30.83% and 10.68% respectively.

### Trends in DDC for different classes of antidiabetic drugs

For the different classes of antidiabetic drugs, the top three drug classes in terms of DDC were GLP-1RAs (¥12.81), combinations of oral blood glucose lowering drugs (¥12.66), insulins and analogues (¥11.79), and the bottom three were sulfonylureas (¥1.17), biguanides (¥1.80) and SGLT2i (¥2.58), with the highest class having an average annual DDC of about 11 times that of the lowest class.

The mean DDC of all antidiabetic drugs showed a decreasing trend over the study period (*B* = -0.0250, *P* = 0.044), with an average decrease of 4 percentage points per year, and this trend was evident after 2017. The DDC of insulins and analogues increased from year to year (*B* = 0.458, *P* < 0.001), whereas the DDC of the remaining oral antidiabetic drugs showed a decreasing trend, with significant decreases for SGLT2i (-26.64%), α-glucosidase inhibitors (-13.74%) and other blood glucose lowering drugs (-7.23%) (Table 1).

**Table 1:** Trends in DDC for different classes of antidiabetic drugs

	**2012**	**2013**	**2014**	**2015**	**2016**	**2017**	**2018**	**2019**	**2020**	**2021**	***B*(95%*CI*)**
Insulin and analogues	9.66	9.45	9.64	11.06	11.96	12.23	12.05	12.36	12.99	13.43	0.458(0.342to0.574)^*^
Biguanides	1.29	1.24	1.54	1.80	1.93	2.03	2.22	2.47	2.35	0.85	0.060(-0.015to0.194)
Sulfonylureas	1.12	0.99	1.02	1.14	1.36	1.41	1.44	1.46	1.02	0.84	0.005(-0.054to0.064)
Combinations		19.86	24.68	24.17	18.61	22.70	12.07	12.39	11.41	10.73	-1.774(-2.803to-0.745)^*^
α-glucosidase	11.16	11.80	12.37	11.19	10.77	10.72	10.56	10.27	2.87	1.60	-0.976(-1.620to-0332)^*^
Thiazolidinediones	4.36	4.30	4.34	4.62	4.81	4.91	4.32	4.09	4.08	3.98	-0.042(-0.119to-0.034)
DPP-4i						8.82	8.59	8.02	7.84	6.55	-0.592(-0.870to-0.188)^*^
GLP-1RAs							14.40	13.98	11.96	12.53	
SGLT2i							8.26	8.41	2.62	2.28	
Other	6.90	6.28	6.70	6.45	6.52	6.59	5.75	5.49	5.42	3.10	-0.293(-0.475to-0.110)
Total	6.51	6.89	7.51	7.78	8.19	8.26	6.49	6.48	5.24	4.20	-0.250(-0.532to-0.031)^*^

*DDC* defined daily cost, *CI* confidence interval, *DPP-4i* dipeptidy1 peptidase 4 inhibitors, *GLP-1RAs* glucagon-like peptide-1 receptor analogues, *SGLT2i* sodium-glucose co-transporter 2 inhibitors

^*^P < 0.05; regression coefficient described trends showing the average annual changes

### The impact of pharmaceutical policy on drug use and expenditure

#### Based on the EMs perspective

**Fig. 3:**
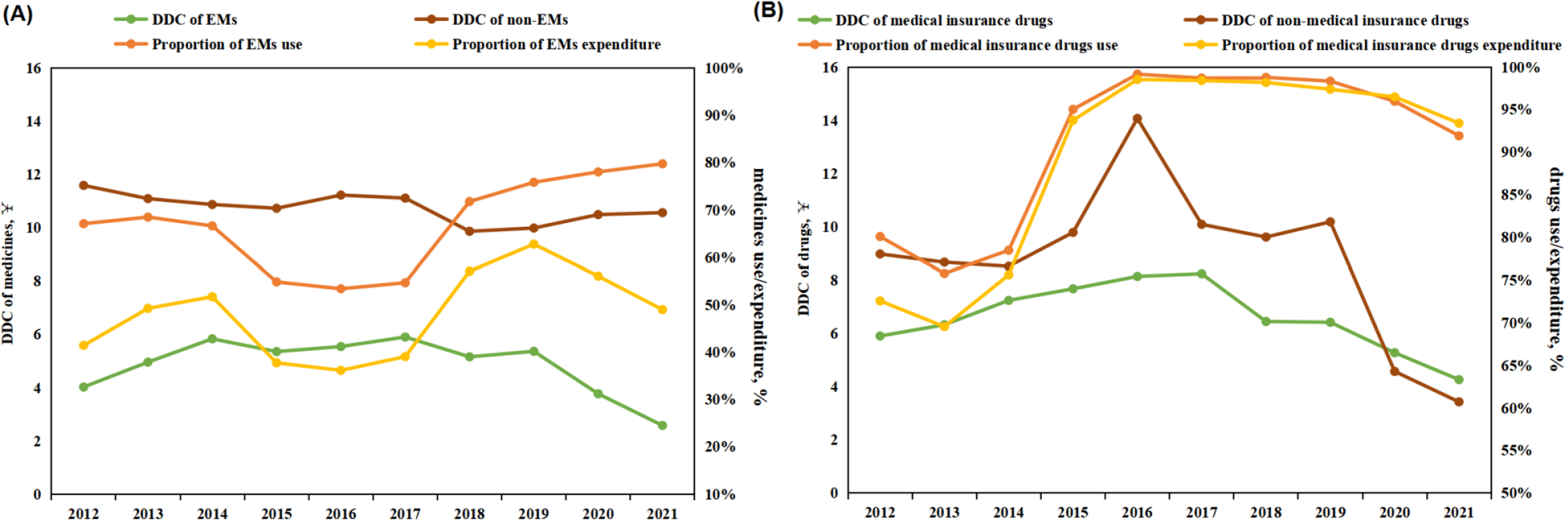
Trends in the use and DDC of EMs and medical insurance drugs. **A** trends in the use and DDC of EMs and non-EMs; **B** trends in the use and DDC of medical insurance and non-medical insurance drugs. DDC, defined daily cost; EMs, essential medicines

The overall trend in the share of use and expenditure of EMs was slowly increasing during the study period, and the relative ratio with fixed base for both was almost the same (18.88% vs. 18.24%). In terms of DDC for both types of drugs, both EMs and non-EMs showed a decreasing trend, but the decrease for EMs was significantly higher than non-EMs (-35.93% vs. -8.84%) (Fig. 3A).

#### Based on the medical insurance classification perspective

Both the use and expenditure shares of medical insurance drugs tended to increase during the study period (*B* = 0.022, 0.030, *P* = 0.024, 0.009), with a significantly higher relative ratio with fixed base in expenditure than in use (28.69% vs. 14.76%). In terms of DDC for both types of drugs, both medical insurance and non-medical insurance drugs showed a decreasing trend, but the decrease for non-medical insurance drugs was significantly higher than for medical insurance (-61.80% vs. -27.77%) (Fig. 3B).

#### Based on the perspective of the centralised procurement

**Fig. 4:**
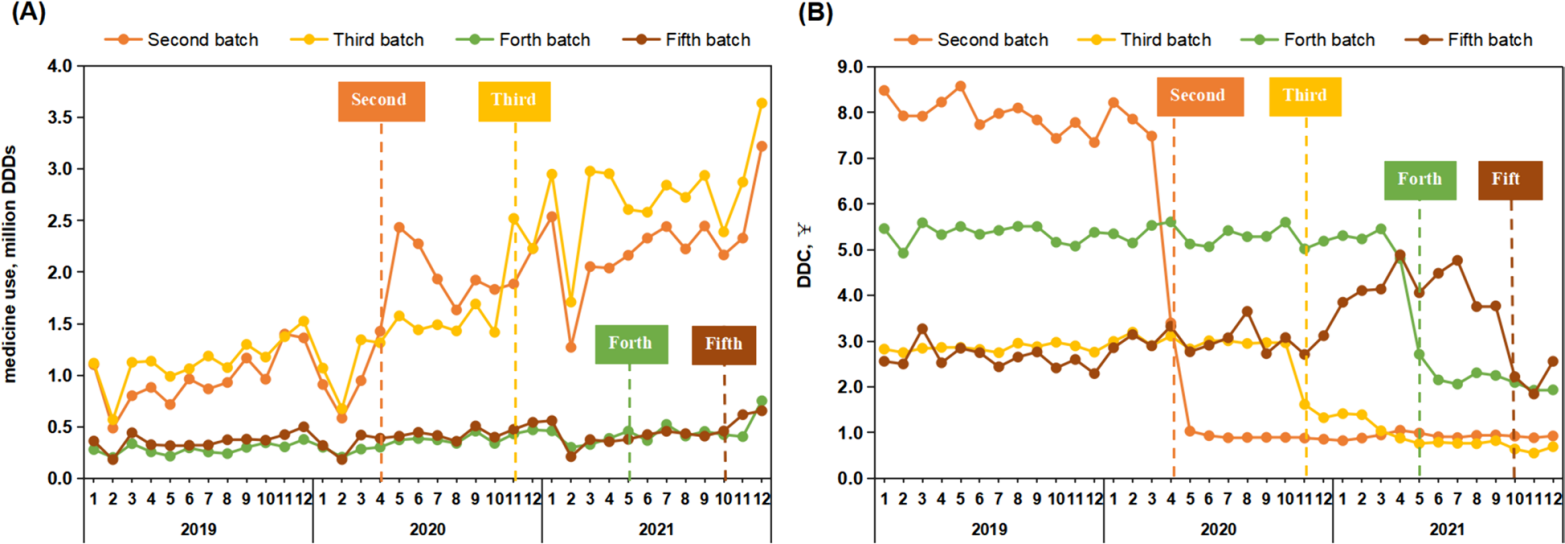
Trends in the use and expenditure of bid-winning drugs under the centralised procurement policy. **A** trends in the use of bid-winning drugs; **B** trends in the expenditure of bid-winning drugs. DDDs, defined daily doses; DDC, defined daily cost

The impact of the centralised procurement policy on the use and expenditure of the bid-winning drugs was found to be accompanied by a short-term response to the implementation of each policy, with a significant increase in use and a significant decrease in expenditure, ultimately manifesting as a "precipitous" decrease in the DDC of the bid-winning drugs. Among them, the second batch of bid-winning drugs had the largest increase in use (189.91%) and expenditure (-76.32%) after the policy, while the impact on the fourth and fifth batches of bid-winning drugs was relatively small (Fig. 4).

In terms of DDC, the price reductions for all batches of bid-winning drugs were above 40%, again with the largest price reduction (81.71%) for the second batch of bid-winning drugs. Among the six antidiabetic drugs with records of use before the implementation of the policy, the top three price reductions were for glimepiride 2mg/30, acarbose 50mg/30 and acarbose 100mg/30, with price reductions of 6.43% (¥1.31), 8.45% (¥11.81) and 9.55% (¥8.81) in that order (Table 2).

**Table 2:** Changes in the DDC of bid-winning drugs before and after the implementation of the centralized procurement policy

Batch	Execution time	Drug name	Specification/ Conversion factor	DDC (¥)		
				**Pre-**	**implementing**	**Post-**
**Second**	24/4/2020	acarbose tablets	50mg/30	12.9	2.95	1.09
			50mg/45		1.08	1.07
			100mg/30	9.74	4.37	0.93
		glimepiride tablets	1mg/30		0.1	0.1
			1mg/60		0.1	0.1
			2mg/30	1.4	0.16	0.09
			2mg/60		0.09	0.08
**Third**	15/11/2020	metformin hydrochloride extended-release tablets	0.5g/60		0.35	0.34
		metformin hydrochloride tablets	0.5g/36		0.26	0.26
		vildagliptin tablets	50mg/20		1.93	
			50mg/30		1.9	1.87
**Forth**	1/5/2021	repaglinide tablets	1mg/30	2.3	1.42	1.41
			1mg/60		1.38	1.38
		gliclazide extended-release tablets	30mg/60		1.22	1.22
		canagliflozin tablets	0.1g/30		3.53	3.53
		nateglinide tablets	60mg/24		3.6	3.6
			60mg/36		3.55	3.55
		empagliflozin tablets	10mg/10		1.84	1.84
			25mg/14			1.47
**Fifth**	15/11/2021	glipizide tablets	5mg/48	0.62	0.32	0.32
		glipizide controlled-release tablets	5mg/14		1.98	1.98
		miglitol tablets	50mg/30	10.76	8.7	8.7
		saxagliptin tablets	5mg/7		2.96	2.96

*DDC* defined daily cost

## Discussion

The global prevalence and incidence of diabetes have risen dramatically since the beginning of the twenty-first century [25]. As diabetes and its early complications were heavily dependent on medication, this changing disease prevalence trend inevitably led to a continued increase in the use of related drugs. In addition, as the level of drug development has improved, new and more expensive antidiabetic drugs with better efficacy and fewer side effects have been included in the recommended treatment guidelines [26] and have been introduced to the market, which have also contributed to a significant increase in drug expenditure. Ultimately, the three main reasons were the ageing of the world's population, the decline in mortality from diseases triggered by improvements in health care, and the decreasing risk factors for disease [27].

Notwithstanding the trends above, the level of use and expenditure on antidiabetic drugs in Gansu Province was much lower than in some economically developed countries and regions worldwide, and was even comparable to that of Denmark at the end of the twentieth century [15, 20, 28, 29]. This is partly because the prevalence rate in Gansu Province was lower than in these countries, and the knowledge and treatment of diabetes are lower, another partly because the consumption of medicines in retail pharmacies was not yet included in our study, resulting in an underestimation of the overall use [30]. To reduce the burden of drugs on the grass-roots population, the relevant departments of the Gansu Provincial Government have in recent years continued to update the medical insurance policy for the two diseases, including the expansion of the scope of outpatient medication services, the upward adjustment of the overall reimbursement rate, the implementation of long-term prescription management and the implementation of instant settlement at the place of medical treatment, among other things [31]. In the future, publicity efforts should also be further stepped up, and with the help of new technologies such as artificial intelligence, big data and cloud computing, personalised health management, proactive follow-up and lifestyle intervention should be carried out for the key groups of people with chronic diseases, to reduce the waste of medical resources due to the unregulated management and treatment of chronic diseases.

The composition of the use of different classes of antidiabetic drugs in Gansu Province varied greatly. Among the oral antidiabetic drugs, biguanides overtook sulfonylureas to jump to first place, and α-glucosidase inhibitors caught up to become the third most used oral antidiabetic drug. To the present time, as these facts are still true——hypoglycemia is one of the dangerous complications of diabetes. Because sulphonylureas tended to trigger weight gain and hypoglycemia in patients [32], international guidelines preferred metformin, which did not increase the risk of hypoglycemia. This drug was still the most widely used first-line treatment in most countries because it combined safety and cost-effectiveness [33]. And for patients with the limited effect of single drug therapy, multiple combinations of metformin and other oral antidiabetic drugs or injectable drugs were used to control blood glucose. The use of α-glucosidase inhibitors was higher in Gansu Province compared to the United States and some European countries. Due to a diet with carbohydrates as a core food and genetic differences, most Chinese had higher postprandial blood glucose levels than Europeans [34], and α-glucosidase inhibitors were widely used to treat type 2 diabetes in Chinese patients because their mechanism of action was to lower postprandial blood glucose levels by inhibiting the absorption of carbohydrates in the upper small intestine [35].

As a representative of the novel antidiabetic drug group, dapagliflozin was approved for entry into China in March 2017, becoming the first SGLT2i to be marketed in China, and in the same year sitagliptin, which belonged to DPP4i, was added to the NBMIDC (Class B) [36], both of which have contributed to the increased use of this drug group. Of course, another reason could be that the use base in the early days was too small. The proportion of novel antidiabetic drugs in Gansu Province has continued to increase over the past five years, with SGLT2i accounting for the highest proportion (about three-fifths), followed by DPP4i, despite being the first novel antidiabetic drug to enter China. Due to the wide disparity in economic and educational levels between urban and rural residents, the rate of early diagnosis and effective treatment of diabetes patients in rural areas of China was low, and patients often waited until they developed serious complications before receiving treatment. Some studies have shown that the incidence of complications in Chinese diabetes patients was higher than in some high-income countries, especially in cardiovascular complications and kidney damage [37]. SGLT2i had good efficacy in treating these complications of diabetes, and the inclusion of this class of drugs in the NBMIDC has resulted in more significant price reductions, which may be a reasonable explanation for the above phenomenon.

The results of the study showed that diabetes treatment in Gansu Province was still concentrated in secondary and tertiary hospitals, and there was no gradual downward trend toward primary health care. Despite the Chinese government's commitment to managing and treating chronic diseases through the primary health care system over the past few decades, including a significant increase in investment in health care resources and human resources [38], an unexpectedly large number of residents still tended to bypass nearby primary health care facilities in favour of higher-level facilities for the treatment of minor ailments. The reason for this was that residents living in urban areas choose high-level hospitals because of their proximity, while as mentioned earlier, some rural residents can only choose high-level hospitals because of the severity of their illnesses [39].

The DDC of different classes of antidiabetic drugs varied considerably, and there is no doubt that insulins and analogues and novel antidiabetic drugs were generally more expensive, which is consistent with other studies [40]. The price of combinations of oral antihyperglycemic drugs was also high. The combination of metformin and thiazolidinediones was the most used compounded hypoglycemic drug in Gansu Province, followed by and novel antidiabetic drug group, which was the main reason for the high price of this class of drugs. In addition to the limitations of the ATC/DDD evaluation system used in the study for the treatment of combinations of oral blood glucose lowering drugs dosage, which also caused the inflated DDC of this class of drugs to some extent.

The use of antidiabetic drugs in Gansu Province has been shifting towards EMs, while gradually moving towards medically insured drugs, and more high-priced drugs were included in the NBMIDC. This phenomenon has been reported in other studies [41]. Two key aspects of drug policy were pricing and reimbursement. In China, provincial governments set uniform procurement prices for their regions within the national guideline price range based on tenders to ensure reasonable value for money [42]. The inclusion of drugs in the NBMIDC meant that pharmaceutical companies could own the majority of the public hospital market, so companies were often willing to "trade volume for price" [43]. At the same time, high health insurance coverage played a benign role in reducing the financial burden of illness on the population and reducing catastrophic health expenses for families, especially for chronic diseases [44]. For the phenomenon of rapid decline in the price of non-medical insurance drugs, which does not exclude enterprises in the bidding for the sake of immediate interests, and was made regardless of the cost of "irrational price reduction" behaviour [45]. This is not a good trend, in the long run will not only lead to the supply of drugs but also not be conducive to the research and development of innovative drugs.

In 2019, the Chinese government launched the first round of national centralised drug procurement to reduce drug prices and save drug costs through economies of scale [46]. Available studies have shown that the implementation of this policy has significantly reduced the DDC of bid-winning drugs, including some common chronic disease drugs (e.g. antihypertensive drugs) and acute disease drugs (e.g. emergency drugs) [47, 48]. As the results show, the DDC of each batch of bid-winning drugs in Gansu Province has experienced a "precipitous" decline, accompanied by a significant increase in dosage, which showed that patients' medication is generally concentrated in the bid-winning drugs. As the number of bid-winning drugs continues to increase in the future, it will be conducive to improving the overall quality of medication for diabetes patients in China and reducing the burden of medication to a greater extent. However, it is worth noting that most of the drugs have only been procured and used since they were selected, reflecting the strong policy guidance on drug selection, which also placed higher demands on the quality of the winning product.

The study has some limitations. Firstly, the data used in the study were procurement data, which may not directly reflect the actual use of antidiabetic drugs. Secondly, the procurement hospitals included in the study were all public healthcare institutions, so the results can not reflect the contribution of private hospitals and retail pharmacies to the consumption of antidiabetic drugs, even though this was a small proportion. Thirdly, due to the limitations of the data information, we were unable to analyse the relationship between market consumption and patient characteristics or prescriber information.

## Conclusions

The study analysed the changing trends and use patterns of antidiabetic drugs and explored the impact of different pharmaceutical policies on drug use and expenditure. It was found that the use and expenditure of antidiabetic drugs showed a continuous upward trend in the last decade, and the prices of drugs were constantly reduced in the direction of benefiting the public, which was closely related to the increasing prevalence of the disease and the implementation of a series of pharmaceutical policies. The introduction of new antidiabetic drugs and changes in guideline recommendations have both influenced drug use patterns and led to significant increases in drug expenditure. This suggested that prevention of the disease is a key priority to reduce the financial burden on the health care system and individuals. In the future, the capacity of primary healthcare institutions to prevent and manage the disease should be improved as a whole, and the national basic public health service program should be carried out solidly, while at the same time strictly controlling the quality of the bid-winning drugs under the centralized purchasing policy, setting up an information system for the files of drug varieties, and steadily advancing the traceability management of the medicines that have been selected.

## Data Availability

The datasets generated or analysed during the current study are not publicly available due to confidentiality policies but are available from the corresponding author upon reasonable request.

## Abbreviations

CBP: Centralised bidding and purchasing
ATC: Anatomical therapeutic chemistry
DDD: Defined daily dose
DID: DDD per 1000 inhabitants per day
DDC: Defined daily cost
EMs: Essential medicines
WHO: World Health Organization
GDP: Gross domestic product
NEMS: National Essential Medicine System
NBMIDC: National Basic Medical Insurance Drug Catalogue
DPP-4i: Dipeptidyl peptidase 4 inhibitors
GLP-1RAs: Glucagon-like peptide-1 receptor analogue
SGLT2i: Sodium-glucose co-transporter 2 inhibitors
DDDs: Defined daily doses
